# Metal-Free Radical Dendrimers as MRI Contrast Agents for Glioblastoma Diagnosis: *Ex Vivo* and *In Vivo* Approaches

**DOI:** 10.1021/acs.biomac.2c00088

**Published:** 2022-06-24

**Authors:** Songbai Zhang, Vega Lloveras, Silvia Lope-Piedrafita, Pilar Calero-Pérez, Shuang Wu, Ana Paula Candiota, José Vidal-Gancedo

**Affiliations:** † Institut de Ciència de Materials de Barcelona, ICMAB−CSIC; Campus UAB, 08193 Bellaterra, Spain; ‡ CIBER de Bioingeniería, Biomateriales y Nanomedicina, Instituto de Salud Carlos III, Campus UAB, 08913 Bellaterra, Spain; § Servei de Ressonància Magnètica Nuclear, Universitat Autònoma de Barcelona, 08193 Bellaterra, Spain; ∥ Departament de Bioquímica i Biologia Molecular, Unitat de Bioquímica de Biociències, Edifici Cs, Universitat Autònoma de Barcelona, 08193 Bellaterra, Spain; ⊥ Institut de Biotecnologia i de Biomedicina (IBB), Universitat Autònoma de Barcelona, 08193 Bellaterra, Spain

## Abstract

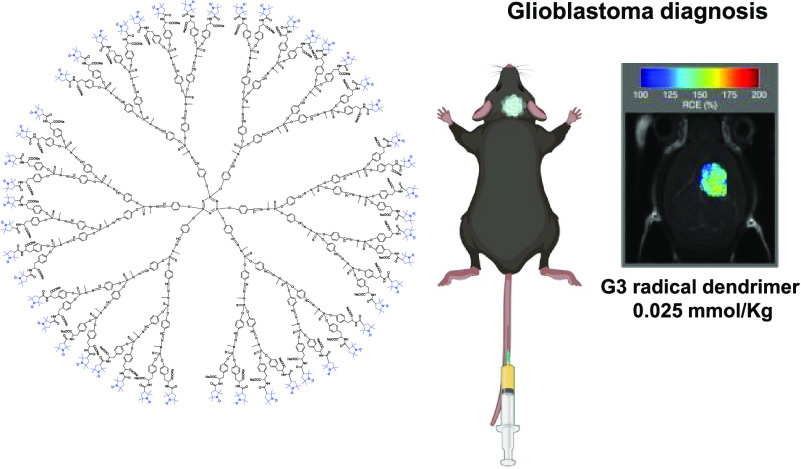

Simultaneously being a nonradiative and noninvasive technique makes magnetic resonance imaging (MRI) one of the highly required imaging approaches for the early diagnosis and follow-up of tumors, specifically for brain cancer. Paramagnetic gadolinium (Gd)-based contrast agents (CAs) are the most widely used ones in brain MRI acquisitions with special interest when assessing blood–brain barrier (BBB) integrity, a characteristic of high-grade tumors. However, alternatives to Gd-based contrast agents (CAs) are highly required to overcome their established toxicity. Organic radicals anchored on a dendrimer macromolecule surface (radical dendrimers) are promising alternatives since they also exhibit paramagnetic properties and can act as *T*_1_ CAs like Gd-based CAs while being organic species (mitigating concerns about toxic metal accumulation). Here, we studied the third generation of a water-soluble family of poly(phosphorhydrazone) radical dendrimers, with 48 PROXYL radical units anchored on their branches, exploring their potential of *ex vivo* and *in vivo* contrast enhancement in brain tumors (in particular, of immunocompetent, orthotopic GL261 murine glioblastoma (GB)). Remarkably, this radical species provides suitable contrast enhancement on murine GL261 GB tumors, which was comparable to that of commercial Gd-based CAs (at standard dose 0.1 mmol/kg), even at its 4 times lower administered dose (0.025 mmol/kg). Importantly, no signs of toxicity were detected *in vivo*. In addition, it showed a selective accumulation in brain tumor tissues, exhibiting longer retention within the tumor, which allows performing imaging acquisition over longer time frames (≥2.5 h) as opposed to Gd chelates. Finally, we observed high stability of the radicals in biological media, on the order of hours instead of minutes, characteristic of the isolated radicals. All of these features allow us to suggest that the G3-Tyr-PROXYL-ONa radical dendrimer could be a viable alternative to metal-based MRI contrast agents, particularly on MRI analysis of GB, representing, to the best of our knowledge, the first case of organic radical species used for this purpose and one of the very few examples of these types of radical species working as MRI CAs *in vivo*.

## Introduction

Magnetic resonance imaging (MRI) is one of the most versatile and widely used clinical diagnostic tools nowadays. MRI provides images of soft tissue anatomy in excellent detail, with high spatial resolution, unlimited penetration depth, long effective imaging window, rapid *in vivo* imaging acquisition, and the absence of ionizing radiation. Accordingly, MRI is largely used for noninvasive diagnosis of tumors, as well as following-up response to therapy or relapse in several organs, but it is especially useful in brain tumors. Brain cancer is one of the most lethal and difficult-to-treat cancers. Surgical resection is the main approach for tumor mass reduction in brain cancer treatment; thus, it is of paramount importance in precise tumor localization and delineation.

Gadolinium (Gd)-based contrast agents (CAs) are the most widely used ones in brain MRI acquisitions for improving the intrinsic contrast enhancement and assessing blood–brain barrier (BBB) integrity, which is a relevant feature in aggressive brain tumors such as glioblastomas (GBs), a high-grade glial tumor with overall survival below 18 months.

Gd-based CAs are categorized as mainly *T*_1_ CAs, also referred to as “positive” contrast agents, leading to an increased (brighter) signal in *T*_1_-weighted images. They present high relaxivities due to the high spin of the paramagnetic Gd(III) ion, which possesses seven unpaired electrons (spin 7/2).^1^ These CAs have historically been considered safe, but for more than a decade, they have been associated with potentially lethal nephrogenic systemic fibrosis.^2−4^ Moreover, recent reports have emerged regarding the accumulation of residual toxic Gd(III) ions in the brain and other organs from patients with an intact blood–brain barrier after the administration of such Gd-based CAs.^5,6^ Since the use of CAs as cancer diagnosis agents in MRI is essential for cancer treatment, in particular for glioblastoma, it is critical to find alternative imaging probes that provide the same or even better paramagnetic properties of current Gd-based CAs.

Nitroxides (or nitroxyl radicals) such as PROXYL or (2,2,6,6-tetramethylpiperidin-1-yl)oxyl (TEMPO) radicals are stable organic paramagnetic species possessing unpaired electrons having the ability to provide imaging contrast by shortening the *T*_1_ relaxation of water in a manner analogous to paramagnetic Gd^3+^. In fact, they have been investigated as *T*_1_ CAs for MRI^7−10^ and have been shown to be nontoxic *in vivo*.^11,12^ However, they have two major limitations. On the one hand, nitroxides present inherent low water ^1^H relaxivity since they possess only one unpaired electron (spin 1/2), and, on the other hand, they are rapidly reduced *in vivo* (half-lives on the order of minutes) to diamagnetic hydroxylamines, hence losing their contrast ability and making them ineffective as contrast agents shortly after injection.^13−16^

One strategy to achieve higher molecular relaxivity and protection against reduction is through the anchoring of many nitroxide units to a conventional linear polymer or a hyperbranched polymer,^17−21^ where the relatively low relaxivity per nitroxide is multiplied by the number of bounded nitroxides and a protective shield effect can be provided to the radicals. Dendrimers could be excellent scaffold candidates for the anchoring of radicals since they are a specific type of polymers characterized by strict control over their structure, making them nearly perfect monodisperse macromolecules. Besides, they present multifunctionality, globular structure and tunable size (through different generations). It is important to highlight that the control over the size of the dendrimers opens the opportunity to modulate their distribution profile in the body, which is not feasible in the case of Gd chelates. Only few reports describe the functionalization of dendrimers with organic radicals (radical dendrimers), with most of them being devoted to studying their electronic, magnetic, or structural properties and only a few of them devoted to MRI CA applications.^22−24^ Still, very little has been reported about their real behavior *in vivo*, remaining a crucial challenging goal. To the best of our knowledge, there are only two examples of *in vivo* studies using radical dendrimers and very few reports using other types of macromolecular polynitroxides. Despite the low solubility in water of the third-generation poly(propylenimine) (PPI) dendrimers conjugated with nitroxides, their intra-articular administration to rabbit stifle joints produced significant enhancement of the articular cartilage in *T*_1_-weighted images.^22^ On the other hand, biodistribution studies were performed with PPI dendrimers conjugated with spirocyclohexyl nitroxides and poly(ethylene glycol) (PEG) chains, providing selectively enhanced magnetic resonance imaging in mice for over 1 h.^23^ Only more recently, another kind of macromolecular polynitroxide, not based on dendrimers but on polymers or polymeric nanoparticles, has shown interesting properties as MRI CA. For example, nitroxide-functionalized brush-arm star polymer organic radical contrast agents (BASP-ORCAs) have shown extremely high *r*_2_ relaxivity and accumulation in murine subcutaneous tumors (A459 tumor-bearing NCR-NU mice) for a long time following systemic administration.^17^ Linear and cross-linked poly(carboxylate ester) PEG-modified PROXYL systems were used to provide MR imaging contrast enhancement to breast cancer tumors.^18^ In addition, amphiphilic poly(ethylene glycol)-*b*-polycarbonate-based diblock copolymers containing pendant persistent PROXYL radicals were locally administered in the hindlimb muscle of a female C57BL/6J mouse.^18^ Finally, when adult female BALB/c nude mice bearing subcutaneous tumors of HeLa cells in their back were intravenously injected with polyacetylenes containing TEMPO and PEG, the MRI signal intensity significantly increased in the tumor parenchyma.^20^ Nevertheless, none of these studies describe MRI performance in orthotopic glioblastoma.

While dendrimer-based magnetic resonance imaging agents decorated with Gd have been reported for brain cancer,^25^ nothing has been described with metal-free organic water-soluble radical dendrimers.

In some recent works, we have proposed the use of radical dendrimers^26,27^ as a suitable alternative to Gd-based CAs.^24,28^ In general, one of the main drawbacks of organic macromolecules such as dendrimers is their low water solubility, especially for large dendrimer generations. One of our proposals was an innovative strategy to increase water solubility and, at the same time, to obtain a full radical functionalization of branches. By using amino acids as linkers between the dendrimer branches and the radicals we obtained four generations of poly(phosphorhydrazone) (PPH)-based G*n*-Tyr-PROXYL (*n* = 0, 1, 2, 3) radical dendrimers fully soluble in water and completely functionalized with 6, 12, 24, and 48 PROXYL radicals, respectively.^28^ We demonstrated that such radical dendrimers offered the pendant radicals higher stability (*in vitro*) against reduction with ascorbate ions and showed negligible *in vitro* cytotoxicity, demonstrating that they could be excellent candidates to be used as MRI contrast agents suitable for biomedical applications. However, the assessment of their *in vivo* properties such as stability and toxicity along with biodistribution and MRI studies was still lacking on tumor-bearing mice, and this is essential to consider them as plausible alternatives to Gd-based CA.

In the present work, we have explored the *ex vivo* and *in vivo* MRI potential characteristics of the highest generation of that family of radical dendrimers, G3-Tyr-PROXYL, as MRI contrast agents in immunocompetent, orthotopic GL261 murine glioblastoma. The *in vivo* toxicity and stability were also assessed. The G3 generation was the compound of choice for these studies since it showed the highest relaxivity and a larger molecular size. We have synthesized the corresponding sodium salt derivative instead of the previously reported lithium salt derivative to improve biocompatibility and safety.

## Experimental Section

### Synthesis

The synthesis of G3-Tyr-PROXYL-ONa was carried out following the procedure previously described by us, with little modifications.^28^

### G3-Tyr-PROXYL-ONa

Under dark conditions, G3-Tyr-PROXYL-OMe^28^ (110 mg, 4.17 μmol, 1 equiv) was added into a round-bottomed flask equipped with a stir bar and dissolved in 2 mL of tetrahydrofuran (THF). NaOH (129 mg, 3.0 mmol, 720 equiv) was dissolved in 2 mL of Milli-Q water and transferred to the THF solution of G3-Try-PROXYL-OMe. The reaction mixture was allowed to stir at room temperature overnight. Afterward, THF was removed under a vacuum, and the aqueous solution was purified by dialysis (MWCO, 0.1–0.5 kDa) to remove the excess NaOH. The external water was changed after 2, 4, 21, and 46 h. Then, the aqueous solution of the dialysis bag was collected and the water was eliminated by freeze-drying to afford the final product G3-Try-PROXYL-ONa as a pale-yellow solid in 67% yield. The full radical substitution was quantitatively characterized by electron paramagnetic resonance (EPR) and its purity by size exclusion chromatography (SEC) (see the Supporting information).

### Materials and Methods

#### Chemicals

*N*-(*tert*-Butoxycarbonyl)-l-tyrosine methyl ester (Boc-Tyr-OMe), Cs_2_CO_3_, 3-carboxy-PROXYL, 1-[bis(dimethylamino)methylene]-1*H*-1,2,3-triazolo[4,5-*b*]pyridinium 3-oxid hexafluorophosphate (HATU), *N*,*N*-diisopropylethylamine (DIPEA), and NaOH were obtained from Sigma-Aldrich. Trifluoracetic acid (TFA) was obtained from EMD Millipore. CH_2_Cl_2_ and CHCl_3_ were distillated from CaH_2_. Tetrahydrofuran (THF) was distillated from Na/benzophenone. Ultrafiltration was performed on solvent-resistant stirred cells from EMD Millipore with regenerated cellulose membranes (3, 5, and 10 kDa) and dialysis with a dialysis kit (MWCO 100–500 Da, Thermo Fisher Scientific Inc.). Ultrapure water (Milli-Q, EMD Millipore) was used for ultrafiltration (together with high-performance liquid chromatography (HPLC)-grade acetone) and dialysis.

#### Animals

GL261 mouse glioma cells were obtained from the Tumour Bank Repository at the National Cancer Institute (Frederick, Maryland). Cells were checked for the mouse short tandem repeat (STR) profile as well as interspecies contamination. In addition, polymerase chain reaction (PCR) studies were performed to discard mycoplasma and virus presence. All studies involving animals were approved by the local ethics committee (*Comissió d’Ètica en Experimentació Animal i Humana*, CEEAH), according to regional and state legislations (protocol references CEA-OH-9685/CEEAH-3665). Mice were purchased from Charles River Laboratories (l’Abresle, France) and housed at the animal facility of the *Universitat Autònoma de Barcelona*. GL261 tumors were induced in a total of 10C57BL/6 female wild-type (wt) mice by intracranial stereotactic injection of 10^5^ GL261 cells as already described by us,^30^ and the n = 7 mice with most homogeneous tumor volumes were chosen for further studies. Mice were weighed twice a week, and tumor volumes were followed up using *T*_2_-weighted image (*T*_2_w) MRI acquisitions. In addition, wt mice were used for studies such as *ex vivo*, biodistribution and tolerability (described in the corresponding sections). Overall, a total of n = 27 C57BL/6 mice (weighing 19.43 ± 1.46 g, aged 12 weeks) were used in this study.

### Size Exclusion Chromatography (SEC)

Size exclusion chromatography (SEC) analysis was carried out using an Agilent 1260 infinity II liquid chromatography system apparatus equipped with a diode array detector. For the G3-Tyr-PROXYL-ONa dendrimer, a PSS Suprema precolumn (10 μm, 8 × 50 mm^2^) and a PSS Suprema analytical column (10 μm, 100 Å, 8 × 300 mm^2^) were used. LiCl 0.25 mM in water was used as the eluent at a flow rate of 0.35 mL/min at 35 °C. The dendrimer was dissolved in the eluent to reach a final concentration of 1 mg/mL dendrimer and filtered through a 0.2 μm nylon filter before injection.

### Electron Paramagnetic Resonance Spectroscopy (EPR)

Electronic paramagnetic resonance spectroscopy (EPR) spectra were obtained with an X-Band (9.7 GHz) Bruker ELEXSYS 500 spectrometer equipped with a ST8911 microwave cavity, a Bruker variable-temperature unit, a field frequency lock system Bruker ER 033 M and equipped with an NMR Gaussmeter Bruker ER 035 M. The modulation amplitude was kept well below the line width, and the microwave power was well below saturation. All liquid samples were previously degassed with Ar. A quantitative EPR study was performed for G3-Tyr-PROXYL-ONa under the same conditions and at the same concentration as for G0- to G3-Tyr-PROXYL-OLi,^28^ comparing the corresponding double integration value of the EPR spectrum with those of the former ones, resulting in an area matching the full radical substitution. EPR spectra of urine were carried out in a quartz flat cell, and the different organ tissues were analyzed using a quartz tissue cell. Previously, tissue organs were weighed in an analytical balance.

### Endotoxin Determination

The endotoxin determination of the G3-Tyr-PROXYL-ONa sample was performed by the ICTS–NANBIOSIS, more specifically by the U20/FVPR at the Vall d’Hebron Institute of Research (VHIR), using the limulus amebocyte lysate (LAL) chromogenic method (A39553 de Pierce).

### Magnetic Resonance Imaging (MRI) Studies

Magnetic resonance imaging (MRI) studies were carried out at the joint NMR facility of the *Universitat Autònoma de Barcelona* and CIBER-BBN (Cerdanyola del Vallès, Spain), Unit 25 of NANBIOSIS ICTS (https://www.nanbiosis.es/portfolio/u25-nmr-biomedical-application-i/). MRI studies were performed in a 7.0 T horizontal-bore superconducting magnet (BioSpec 70/30; Bruker BioSpin, Ettlingen, Germany) equipped with actively shielded gradients (B-GA12 gradient coil inserted into a B-GA20S gradient system). For mouse brain MRI, a 72 mm inner-diameter linear volume coil was used as the transmitter, and a dedicated mouse brain quadrature surface coil was used as the receiver. For whole-body MRI, a 72 mm inner-diameter quadrature volume coil was used as the transceiver. MR data were acquired and processed on a Linux computer using Paravision 5.1 software (Bruker BioSpin GmbH, Ettlingen, Germany).

Solutions of gadopentetate dimeglumine (Gd-DTPA, Magnevist) and the G3-Tyr-PROXYL-ONa radical dendrimer were prepared in a saline solution (NaCl 0.9%, B. Braun), and the injection volume was adjusted according to mice weight.

### Animal Experimental Design

Tolerability and biodistribution studies were performed in healthy (nontumor-bearing) C57BL/6 female mice. Since contrast-enhancement explorations are not expected to be repeated and cumulative, these studies were performed with single-dose administrations. For biodistribution studies, a 0.00625 mmol/kg dosage was used, with the objective of assessing the main organs related to G3-Tyr-PROXYL-ONa radical dendrimer metabolization through MRI studies, which is not expected to vary for different doses. However, tolerability studies were performed with the same dosage foreseen to be used in the dynamic contrast-enhanced magnetic resonance imaging (DCE-MRI) studies with the objective to ensure that no harmful effect was observed, at least with C57BL/6 healthy female mice.

MRI studies for assessing brain tumor contrast enhancement were performed with GL261 GB-bearing mice. Contrast agent administration (both the G3-Tyr-PROXYL-ONa radical dendrimer and the Gd-based commercial CA) was done intravenously under anesthesia. The MRI exploration was performed, and animals were allowed to recover in a warm environment. Mice were euthanized after the whole procedure was finished.

#### MRI Studies

##### *Ex Vivo* Brain MRI Studies

The CAs for *ex vivo* studies were dissolved in a saline solution (0.9% NaCl, B. Braun). The amount finally used for each mouse was 5 nmol of Gd dissolved in 4 μL of saline solution and 1.25 nmol of G3-Tyr-PROXYL-ONa radical dendrimer. Mice were euthanized by cervical dislocation and immobilized on a stereotactic holder (Kopf Instruments, Tujunga, California). The contrast administration was carried out as described for tumor generation, with three injection points as previously described.^31^ The whole process of CA injection *ex vivo* took 30 min.

All imaging studies started with *T*_2_-weighted MRI screening with high-resolution coronal *T*_2_w images using a rapid acquisition with relaxation enhancement (RARE) sequence to evaluate brain tumor presence and to monitor its evolution stage. The acquisition parameters for MRI studies were as follows: repetition time (TR)/effective echo time (TE_eff_) = 4200:36 ms; echo train length (ETL) = 8; field of view (FOV) = 19.2 × 19.2 mm^2^; matrix size (MTX) = 256 × 256 (75 μm/pixel × 75 μm/pixel); slice thickness (ST) = 0.5 mm; inter-ST = 0.1 mm; number of slices (NS) = 10; number of averages (NA) = 4; and total acquisition time (TAT) = 6 min and 43 s.

*T*_1_-weighted MRI: A multispin–multiecho (MSME) sequence was used with FOV = 17.6 × 17.6 mm^2^; MTX = 128 × 128 matrix (138 μm/pixel × 138 μm/pixel); TR/TE_eff_ = 200:8.5 ms; number of repetitions (NR) = 1; and TAT = 4 min 16 s.

##### *In Vivo* MRI Studies

Mice anesthesia was performed with isoflurane (B. Braun, Melsungen, Germany) at 0.5–1.5% in O_2_, and the respiratory frequency was maintained between 40 and 60 breaths/min. Body temperature was maintained with a recirculating water system incorporated in the animal bed and measured with a rectal probe. Respiration rate and temperature were constantly monitored (SA Instruments, Inc., New York). Before immobilization in the animal holder, each mouse was cannulated in the tail vein using a home-built multidelivery polyethylene tubing system. In this case, a 30G two-way catheter was connected through polyethylene tubing, to two independent 1 mL syringes (Becton–Dickinson S.A., Madrid, Spain) loaded with heparinized-saline (40 U/mL) (0.9% NaCl, B. Braun and heparin, Mayne Pharma España, Madrid, Spain).

##### Brain

The *T*_2_- and *T*_1_-weighted MRI were performed with the same parameters as described for *ex vivo* MRI.

*T*_1_ maps were performed with the RARE-VTR sequence with FOV = 17.6 × 19.28 mm^2^; MTX = 128 × 128 matrix (138 μm/pixel × 150 μm/pixel); Teff = 7.5 ms and TR list: 100, 400, 700, 1000, 1300, 1700, 2000, 2600, 3500, and 5000 ms. NR = 1, TAT = 19 min 31s.

##### DCE-MRI Studies

The contrast agent was injected into the mice as a bolus (72–84 μL, doses varied according to the CA ranging from 0.00625 to 0.1 mmol/kg) during dynamic contrast-enhanced (DCE)-*T*_1_ MRI studies. Three glioma-bearing mice were injected with gadoterate meglumine, and another three were injected with G3-Tyr-PROXYL-ONa. A DCE *T*_1_ study was then performed using three coronal sections. For this, an MSME sequence was used with FOV = 17.6 × 17.6 mm^2^; MTX = 128 × 128 matrix (138 μm/pixel × 138 μm/pixel); TR/TE = 200:8.5 ms; ST = 1 mm; NA = 2; NR = 70; TAT = 59 min 44 s. The contrast bolus was administered after the third repetition of the complete *T*_1_-weighted sequence (about 2.5 min after the start of the image acquisition protocol). DCE-MRI data were analyzed with DCE-@urLAB (http://oa.upm.es/28901/).

##### Body

*T*_1_-weighted images were acquired using a respiratory gated spin echo sequence (repetition time (TR)/echo time (TE) = 600:10.5 ms) acquiring 23 coronal sections with a field of view = 10 × 5 cm^2^, matrix size = 512 × 256, and slice thickness of 1 mm with a 0.1 mm gap between slices

*T*_2_- and *T*_1_-weighted scout images were initially performed to be used as reference images for prescribing the final coronal sections through the main organs of interest (kidneys, liver, bladder, muscle, spleen). Afterward, DCE-*T*_1_w images and *T*_1_ map respiration gated acquisitions were performed before and after contrast agent injection. For whole-body DCE-*T*_1_w, an MSME sequence was used with FOV = 9 × 3 cm^2^; MTX = 256 × 128 matrix; TR/TE = 400:10 ms; NA = 2; ST = 1 mm with 0.2 mm gap between slices; TAT = 1 min 42 s.

*T*_1_ maps were performed on the same sections as DCE-*T*_1_w images using a RARE-VTR sequence with FOV = 9 × 3 cm^2^; MTX = 128 × 128 matrix, TE = 7.5 ms; and TR list: 250, 400, 800, 1300, 1700, 2400, and 3500 ms. NR = 1; TAT = 11 min 2 s. *T*_1_ map acquisitions were used to calculate *T*_1_ values, drawing ROIs in defined zones and measuring the estimated *T*_1_ before and after CA administration, further expressed as a percentage change.

##### Data Availability

The raw/processed data required to reproduce these findings will be available under reasonable request to corresponding author(s).

## Results and Discussion

### Synthesis

First, we synthesized the G3-Tyr-PROXYL-OMe radical dendrimer derivative following the procedure already described in the literature by us.^28^

**Figure 1:**
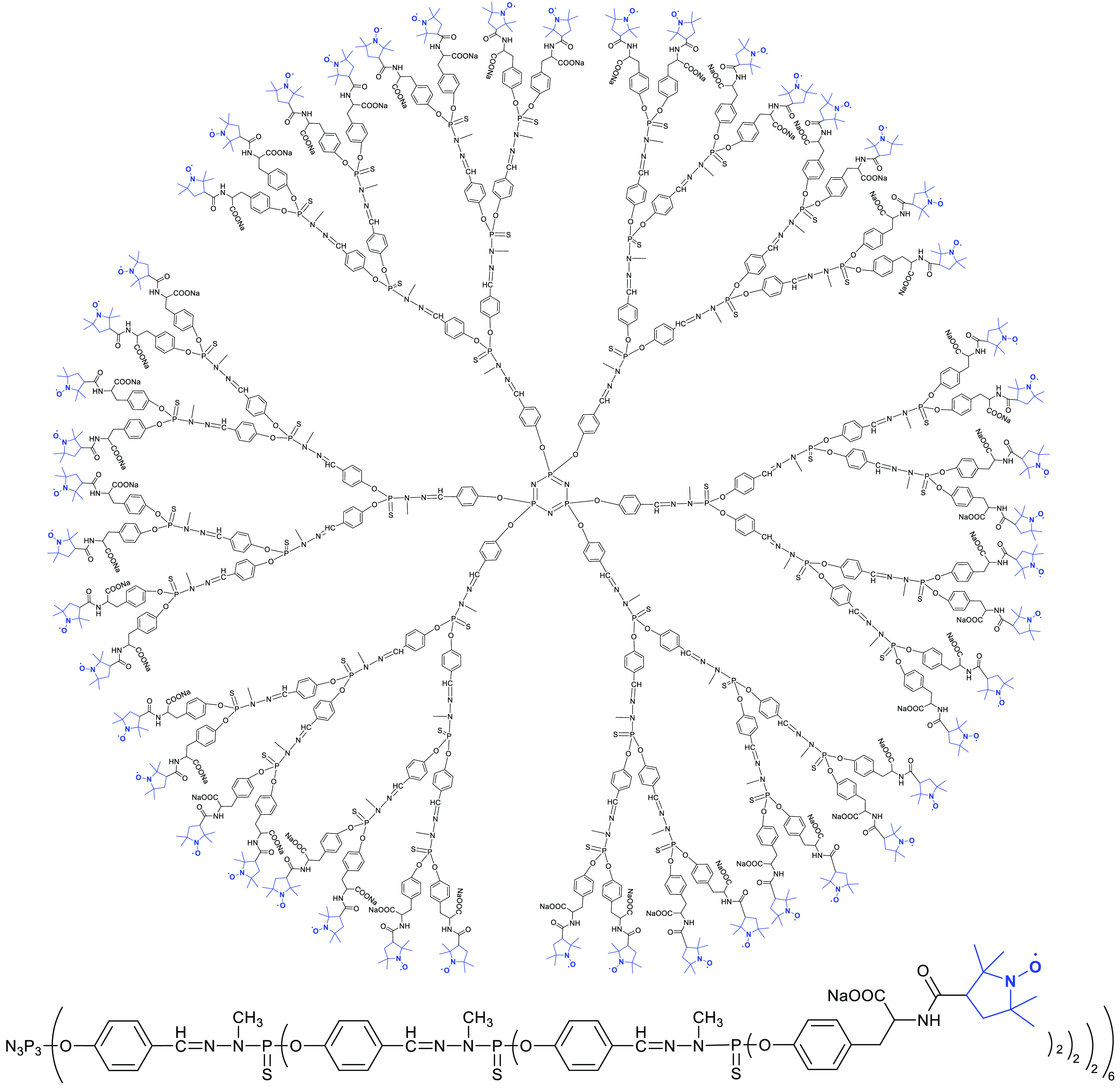
Structure of the G3-Tyr-PROXYL-ONa radical dendrimer.

Then, the methyl ester was hydrolyzed with NaOH in THF/H_2_O (1:1), resulting in the water-soluble G3-Tyr-PROXYL-ONa dendrimer. The excess NaOH was removed by dialysis to purify the G3-Tyr-PROXYL-ONa dendrimer, henceforth referred to as the G3 radical dendrimer (Figure 1). The full functionalization of the G3 dendrimer with radicals was confirmed by EPR, and its purity was monitored by SEC (see the Supporting Information).

### *Ex Vivo* MRI Analysis

**Figure 2:**
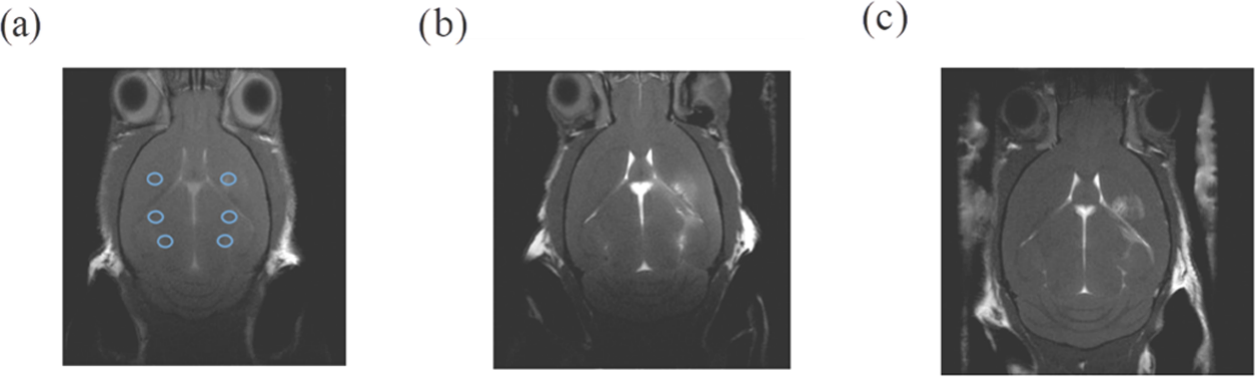
(a) Schematic representation of the region of interest (ROI) selection (blue circles) for analysis in *T*_1_-weighted MRI, indicating ipsilateral ROIs where CA are injected (right) and contralateral ROIs (left). *T*_1_-weighted MRI after *ex vivo* administration of (b) 5 nmol of gadopentetate dimeglumine to each injection point and (c) 1.25 nmol of the G3 radical dendrimer at each injection point.

We followed an *ex vivo* method developed to select CAs with a suitable potential for *in vivo* efficiency using small amounts of compounds and minimum animal use.^31^ Note that these analyses are not intended to set the amount of CA to be administered *in vivo* but rather to investigate whether some aspects of the tumor environment (not possible to be evaluated *in vitro*) could modify/modulate the ability of CA to produce contrast enhancement. A setup experiment with gadopentetate dimeglumine was performed for comparison purposes, using 5 nmol of gadopentetate dimeglumine to each injection point in the brain parenchyma. The relative contrast enhancement (RCE) was calculated, achieving a value of 232% ± 29, *n* = 3, and in agreement with previous *ex vivo* experiments with similar Gd-based contrast agents. The resulting *T*_1_-weighted MRI is shown in Figure 2b.

After a first trial using 0.1 nmol of the G3 radical dendrimer, which only produced a faint relative contrast enhancement, the final administered amount that produced a noticeable relative contrast enhancement in *T*_1_w MRI was found to be 1.25 nmol of G3. With this administered amount, a clear enhancement was observed (Figure 2c). The corresponding calculated relative contrast enhancement was 237% ± 40, *n* = 3, which meant no significant differences compared with RCE calculated for 5 nmol Gd, suggesting that this concentration was enough to get comparable RCE, at least in the *ex vivo* environment.

**Figure 3:**
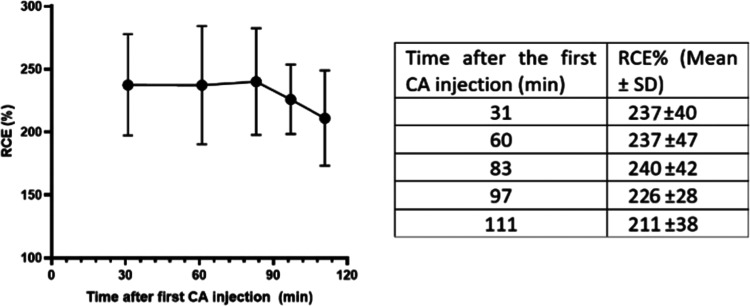
RCE% time-course curve (left) and data (right) obtained from the quantification of *T*_1_w MRI images. The curve displays the average RCE obtained along the time after *ex vivo* administration of 1.25 nmol G3 radical dendrimer. Bars show ± SD.

We also investigated whether RCE presented significant changes along time due to possible radical inactivation. This was a first attempt to assess the stability of the radical dendrimer in a real biological medium, with a view to its use *in vivo*. The first *T*_1_w MRI was acquired 31 min after injection (the shorter timeframe feasible considering the experimental setup). In this first acquisition, the measured RCE was 237% ± 40. After this first *T*_1_w acquisition, quantitative *T*_1_ maps were acquired, followed by sequential *T*_1_w MRI until 111 min post the first injection (Figure S2), and RCE values were accordingly measured (Figure 3). Although the values tended to be slightly lower in the last time points analyzed, the overall RCE variation did not present statistical significance when the different time points were compared (*p* always > 0.05). Thus, the stability of the radicals anchored to the dendrimer scaffold seemed quite prolonged in time in such a biological medium, presenting a similar RCE well beyond 1 h after injection. These data were encouraging for its subsequent use *in vivo*, since, as explained above, one of the main limitations of nitroxides is their rapid bioreduction.

*T*_1_ values after the G3 radical dendrimer local injection were also assessed, and significant changes were detected. Ipsilateral (injection) ROIs presented an estimated *T*_1_ of 1076 ± 320 ms, whereas contralateral ROIs (control) presented values of 1914 ± 23 ms, with a significant average decrease of 46%, in agreement with satisfactory results obtained for RCE measurements in *T*_1_w MRI. Examples of curve adjustments for *T*_1_ maps are shown in Figure S3.

### Endotoxin Analysis of the G3 Radical Dendrimer

Before *in vivo* studies, an endotoxin analysis of the G3 radical dendrimer was carried out to confirm the absence of such toxin in samples administered to mice. We performed the analysis at two different concentrations: 0.4 and 4 mg/mL. In the sample with a lower concentration, the value obtained (0.02 EU/mL) was below the detection range, and in the sample with a higher concentration, very low endotoxin levels were detected: 0.034 ± 0.003 EU/mL. These levels were considered acceptable since the limit for administration in mice would be below 1 EU/mL (5 EU/kg for the usual routes of administration).

### *In Vivo* Studies

In an attempt to use the minimum amount of the G3 radical dendrimer in the *in vivo* studies and produce as similar as possible a Gd-like enhancement, we used two types of concentrations: first, a lower one of 0.00625 mmol/kg; and later, a higher one (0.025 mmol/kg). These concentrations were chosen taking into account the usual Gd-based CA dose used in clinical and preclinical approaches (0.1 mmol/kg) and the *in vitro* relaxivity of the G3-Tyr-PROXYL-ONa radical dendrimer: *r*_1_ relaxivity measured at 7 T (13 s^–1^·mM^–1^) was on the order of 4 times higher than Gd-DTPA relaxivity (3.2 s^–1^·mM^–1^),^29^ similar to the lithium salt derivative.^28^

The lower dose (0.00625 mmol/kg) of the G3 dendrimer is 16 times lower than the usual Gd-based CA dose (0.1 mmol/kg), but taking into account the 4 times higher relaxivity of G3, we could expect only “4 times lower enhancement” than with Gd-based CA. Thus, it could be a good starting point. In fact, this dose proved suitable to determine properly the biodistribution due to reasonable enhancement detection. However, it proved inappropriate for tumor detection since it produced only a slight enhancement in the tumor periphery.

On the other hand, the higher dose chosen later (0.025 mmol/kg) is 4 times lower than the standard dose of Gd-based CA (0.1 mmol/kg), but “in terms of enhancement”, we could expect similar enhancement as Gd-based CA taking into account the aforementioned relaxivity values. In fact, this dose proved suitable for tumor detection.

### *In Vivo* Biodistribution and Tolerability

The preliminary biodistribution studies (MRI-based) were performed with the G3 radical dendrimer at 0.00625 mmol/kg (lower dose) administered through the tail vein, with *n* = 3 wt female C57BL/6 mice. With this experiment, we aimed to assess any *T*_1_ changes in different organs to check for the biodistribution of G3 after intravenous administration.

**Figure 4:**
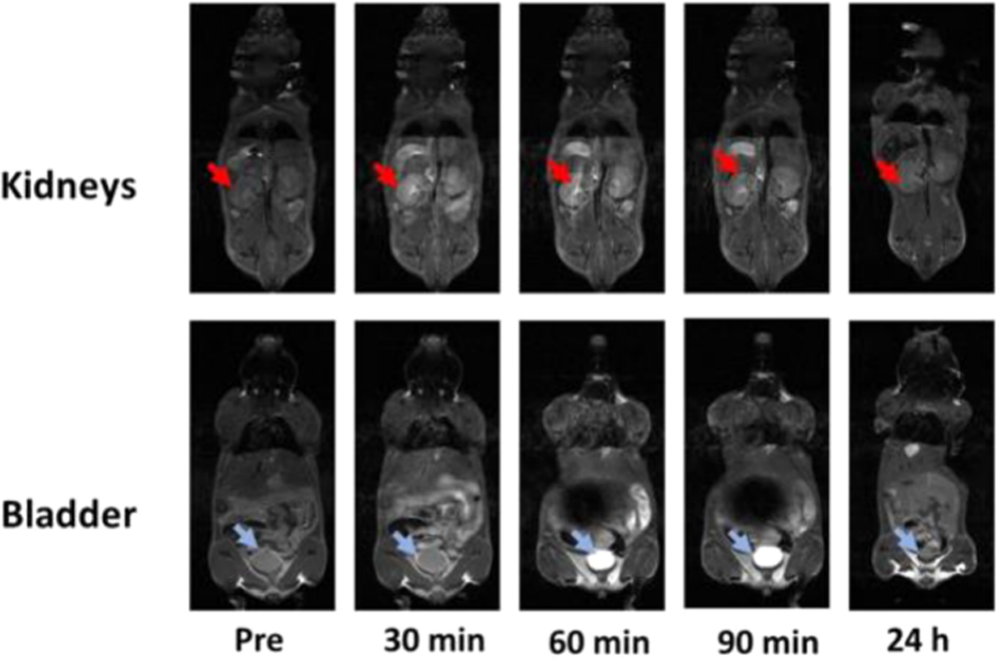
*T*_1_w MRI at different times after G3 radical dendrimer administration (0.00625 mmol/kg). *T*_1_w representative images showing a slice through the kidneys (upper row) and through the bladder (lower row). The main enhancement was seen in the kidney cortex/pelvis and bladder.

The G3 radical dendrimer administration produced a slight enhancement in different organs of wt mice on whole-body *T*_1_w MRI (Figure 4), confirmed by a decrease in *T*_1_ values calculated in *T*_1_ maps. The corresponding *T*_1_w enhancement was mostly observed in the kidney cortex and pelvis. Kidney *T*_1_ decreased by 37.7% at 20 min and by 23.8% at 60 min postadministration, which corresponded to 41 and 16% overall increases in the *T*_1_w signal intensity, suggesting the relevance of renal excretion for these compounds. This was further confirmed by data obtained from the bladder: *T*_1_ decrease of ca. 36% and signal intensity increase of 84%.

The biodistribution data for the higher dose finally chosen for MRI studies was expected to be fully comparable to the lower dose. For confirmation purposes, one GL261 GB-bearing mouse was explored with whole-body MRI 3 h after G3 radical dendrimer administration at 0.025 mmol/kg once DCE-MRI studies were finished. Although the timing did not completely match, the main contrast enhancement and *T*_1_ value decrease were also seen in the kidney cortex and pelvis (41–59% in comparison with the basal measurements in wt mice) and bladder (95% decrease), which is even higher than the values found in the biodistribution study with the lower dose. Since the main biodistribution parameters were confirmed, we considered that the previous biodistribution experiment produced enough signal to be analyzed and was not repeated in a whole cohort of animals with the increased dose. Moreover, biodistribution obtained from EPR data at lower and higher doses (see the next section) was in agreement with the data obtained from whole-body MRI acquisitions.

A tolerability study was performed on three healthy C57BL/6 female mice administered with the higher dose 0.025 mmol/kg G3 radical dendrimer and closely followed up 10 days after injection since acute toxicity would show up in the first few hours/days. In addition, they were also inspected for health status and welfare for a month.

**Figure 5:**
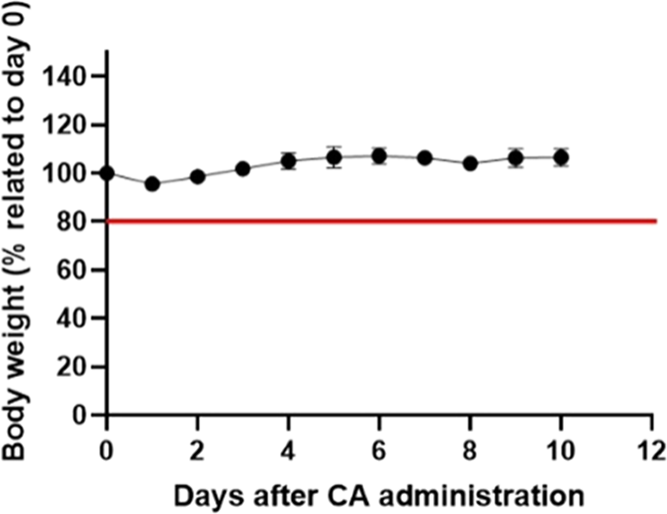
Percentage of body-weight variation of mice injected with the G3 radical dendrimer at dose 0.025 mmol/kg in the G3 dendrimer. The red line indicates the 20% weight reduction point. Average starting values and body-weight evolution are in agreement with values described from the Jackson Laboratory (https://www.jax.org/jax-mice-and-services/strain-data-sheet-pages/body-weight-chart-000664).

Administered mice did not show toxicity symptoms and did not experience body-weight loss either during the first 10 days or in the whole month of the follow-up. Figure 5 shows the variation of weight along time. Their weight evolution was as expected according to the Charles River growth chart for healthy C57BL/6 females of this age. Their overall states such as fur aspect, hydration, and behavior/activity were satisfactory. These results demonstrate the nontoxicity of the G3 radical dendrimer *in vivo* after systemic intravenous tail-vein injection at this dose.

### EPR-Based Biodistribution and Radical Stability *In Vivo*

Mice administered with the G3 radical dendrimer at lower and higher doses were euthanized to check the amount of G3 in some organs by electron paramagnetic resonance (EPR) spectroscopy. A similar mass of organ tissues was analyzed to obtain comparable results, and no significant differences were found between the lower and higher doses’ results.

**Figure 6:**
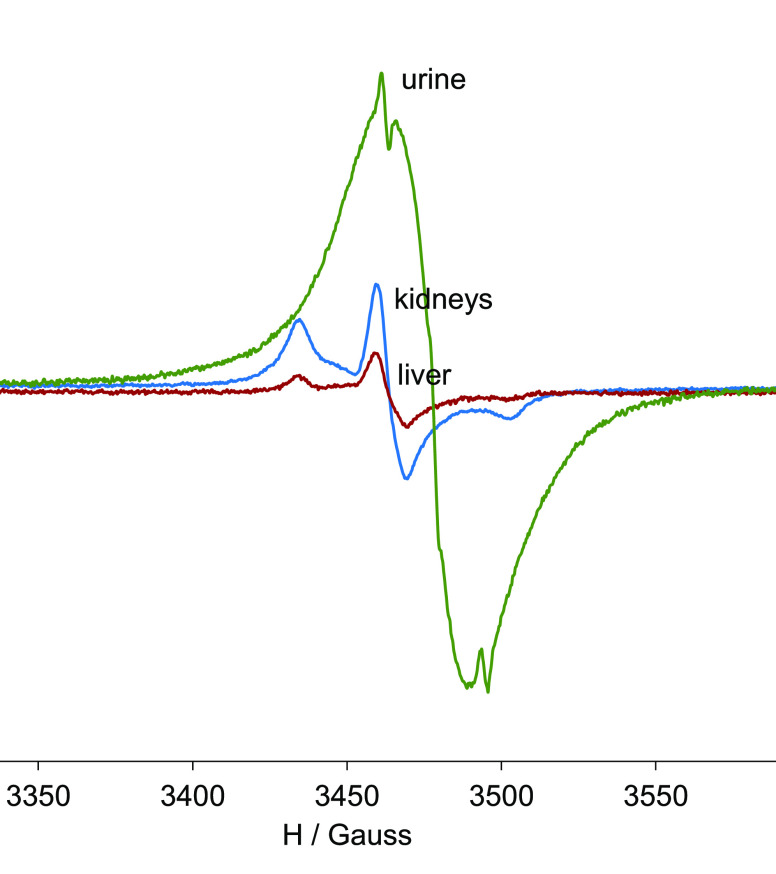
EPR spectra of urine, kidneys and liver from a C57BL/6 female wt mouse administered with 0.00625 mmol/Kg of G3-Tyr-PROXYL-ONa radical dendrimer, euthanized 1.5 h after of intravenous administration. The urine spectrum was obtained from the collected urine diluted to 1:2 with miliQ water.

We analyzed the urine, kidneys and liver of a C57BL/6 female wt mouse administered with 0.00625 mmol/kg (see Figure 6 and the Supporting Information for additional data). The highest amount of the G3 radical dendrimer (highest EPR signal intensity) was found in urine (even taking into account that it was diluted 1:2 with miliQ water to get the optimum volume to be properly measured), followed by kidneys and liver. This result was in agreement with the whole-body *T*_1_w MRI, also suggesting excretion of the radical dendrimer through the kidneys. Interestingly, the EPR spectrum shape of urine sample was almost identical to the G3 spectrum before injection (see Figure S4). This means that the radical character of PROXYL radicals in the G3 radical dendrimer was not quenched by circulating in the bloodstream, passing from the blood to the kidneys and the bladder, 1.5 h postadministration. This is a relevant result that demonstrates the high stability of the radicals when anchored to the dendrimer, in contrast to the fast reduction *in vivo* experienced by isolated nitroxides, especially in the bloodstream and tissues, losing their contrast ability shortly after injection.^13−16^

We also analyzed the kidneys, liver, brain tumor, healthy brain, and muscle of a GL261-tumor-bearing mouse administered with the same dose 0.00625 mmol/kg 1.5 h postinjection (see Figure S5), showing similar results. The largest amount of the G3 radical dendrimer was found in the kidneys, followed by the liver and the brain tumor, while an almost imperceptible EPR signal was detected in the healthy brain and muscle. It is worth mentioning that an important amount of the G3 radical dendrimer was detected in the brain tumor, while the healthy surrounding brain barely showed any G3 signal, suggesting that the G3 radical dendrimer can selectively accumulate in tumors with few spreading to the surrounding brain. This is a relevant and desirable characteristic in a contrast agent intended to be used for brain tumors.

**Figure 7:**
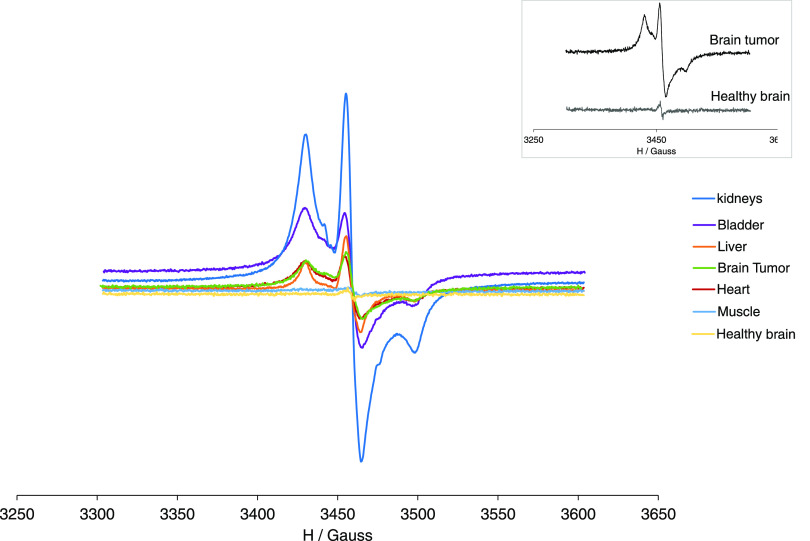
EPR spectra of kidneys, bladder, liver, heart, muscle, brain tumor, and contralateral healthy brain from GL261-tumor-bearing mice after 15 h of 0.025 mmol/kg administration of the radical dendrimer.

In addition, a GL261 GB-bearing mouse with 0.025 mmol/kg dose was euthanized 15 h after administration, and EPR was performed on bladder, kidneys, liver, heart, brain tumor, healthy brain, and muscle. Similar organ tissue mass was analyzed by EPR (around 52 mg each), and the corresponding spectra are plotted in Figure 7. Similar to what was obtained with the lower dose administered, the largest EPR signal intensity was found in the kidneys, followed by the bladder, liver, brain tumor, and heart. It is also important to remark that the selective accumulation behavior in the brain tumor was reproduced at a higher dose (see the inset in Figure 7).

The observation of an intense EPR signal after 15 h of intravenous administration confirmed the stability of PROXYL radicals *in vivo*, farther beyond that of isolated nitroxides. Related to this, the EPR signal observed in the heart means a long circulation half-life of the radical dendrimer.

### *In Vivo* MRI Studies with GL261 GB-Bearing Mice

As previously mentioned, the lower dose of the G3 radical dendrimer (0.00625 mmol/kg) administered to GL261 GB-bearing mice only produced a slight enhancement in the tumor periphery and some hot spots within the tumor (Figure S6), which may correspond to more highly perfused regions. A whole set of mouse brain MRI studies, including DCE-MRI, was performed, as described in the Experimental Section.

**Figure 8:**
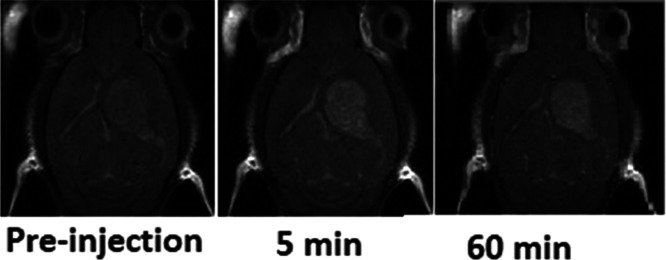
Top: Axial *T*_1_w MRI for the follow-up of tumor contrast enhancement before and after 5 min (ca. maximum enhancement) and 60 min (final of first DCE-*T*_1_w-MRI follow-up, refer to Figure 9) of G3 radical dendrimer administration (0.025 mmol/kg) to GL261 GB-bearing mice.

However, importantly, the higher dose (0.025 mmol/kg) produced a noticeable RCE in the tumor (Figures 8 and S7). The aforementioned G3 radical dendrimer dose was intravenously injected to *n* = 3 GL261 GB-bearing mice, and experiments were conducted as for the lower dose previously described.

Changes observed in *T*_1_ values were mostly seen in tumors rather than in the contralateral brain. *T*_1_ values were calculated before and after G3 administration with *T*_1_ map sequences ca. 1 h after G3 administration. *T*_1_ decrease in the tumor was 19.2% (29.8% increase in the measured signal), while no noticeable changes were found in the corresponding contralateral part.

The RCE and the kinetics of uptake and washout of the G3 radical dendrimer at 0.025 mmol/kg dose were compared with Gd-based CA administration at the standard dose of 0.1 mmol/kg, as well as for 0.04 mmol/kg. Only the first 60 min in the first DCE-MRI experiment of G3 were used for comparison with Gd.

**Figure 9:**
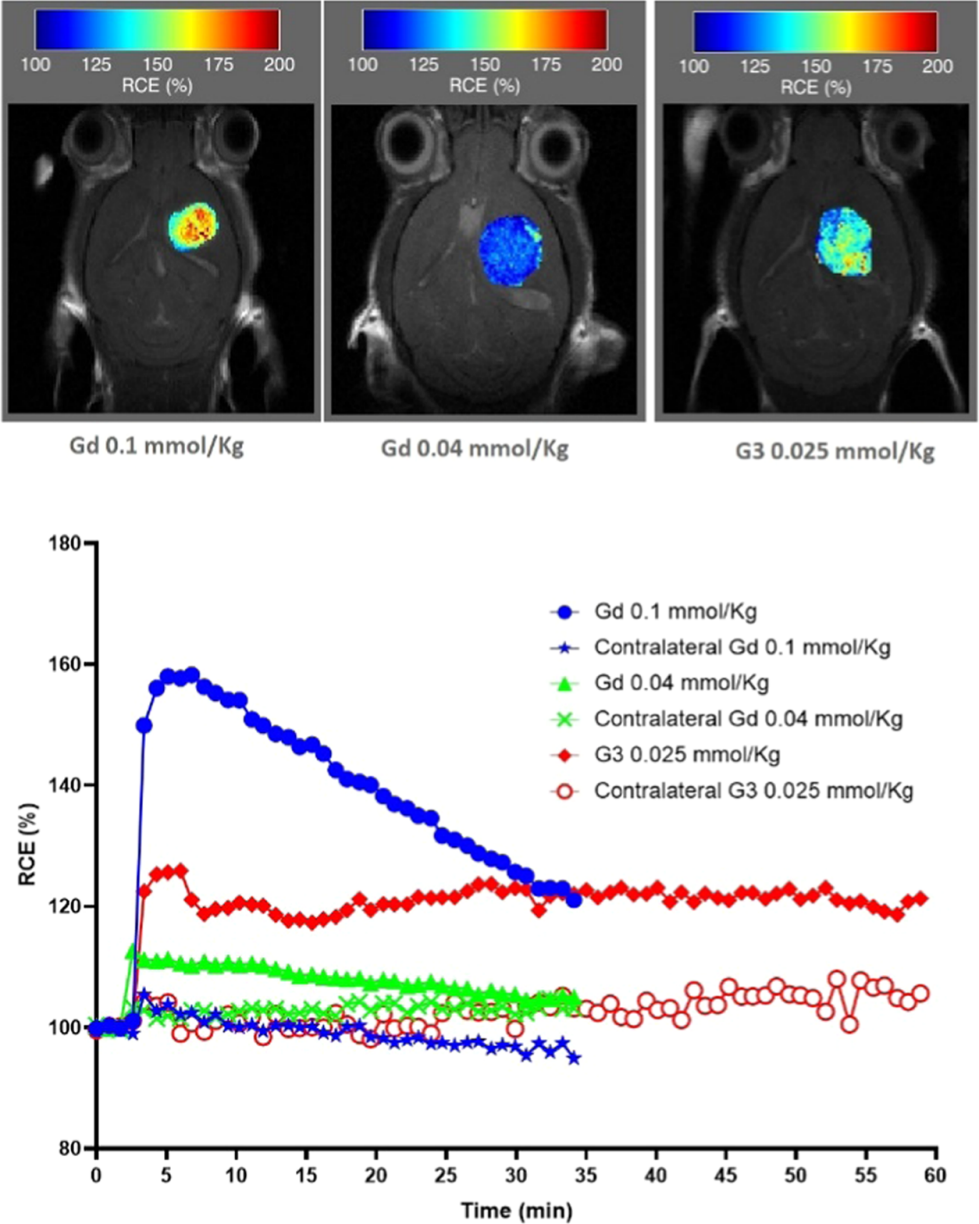
Top: Color-code scale for RCE (Gd 0.1 and 0.04 mmol/kg, 30 min; and G3 radical dendrimer 0.025 mmol/kg, 60 min). Bottom: ROI kinetics for these agents, showing the slower washout of G3. The maximum RCE calculated was 158% for Gd 0.1 mmol/kg, 113% for Gd 0.04 mmol/kg, and 126% for G3 0.025 mmol/kg. Contralateral enhancement was reproducible in all cases (102 ± 3%). Please note that 0.04 mmol/kg administration data comes from retrospective cases administered with Gd-DOTA instead of Gd-DTPA. Analyses were performed with the DCE@urLAB software package.^32^

Remarkably, the RCE measured for the G3 radical dendrimer at 0.025 mmol/kg dose was similar to, although slightly lower than, the RCE obtained with Gd-DTPA at the standard dose of 0.1 mmol/kg and proved higher than the RCE obtained with Gd at 0.04 mmol/kg (Figure 9). However, interestingly, the kinetics of washout was completely different between both contrast agents. The maximum contrast enhancement with Gd-DTPA varied with dose, ranging from 113 to 158%, and started to decrease sharply after the first 5–6 min, suggesting a fast washout. The RCE decreased to 125% after 30 min in the case of the 0.1 mmol/kg dose and recovering basal values in the case of the 0.04 mmol/kg dose. On the other hand, enhancement achieved with G3 radical dendrimer administration proved mostly sustained along the time measured. The enhancement of the tumor zone reached a maximum of 126% after 6 min and remained essentially unchanged during the whole time course (RCE of 121% after 60 min). RCE data suggests that *T*_1_ contrast enhancement was sustained as long as 2.5 h after CA administration (not shown). Therefore, the measured data suggest that tissue enhancement triggered by the G3 radical dendrimer administration may persist well beyond the washout time of Gd.

This is also a relevant point to highlight, suggesting that an improvement in the imaging time frames could be achieved with G3 radical dendrimer administration when compared to Gd chelates, which show a very fast clearance.

In summary, the selective accumulation behavior of the G3 radical dendrimer in the brain tumor, the high stability of the radicals *in vivo*, and the long circulation half-life of the radical dendrimer make G3 radical dendrimers capable of imaging brain tumors over clinically meaningful time scales following systemic administration, at longer time periods than Gd chelates, without concerns over long-term tissue accumulation of metals.

## Conclusions

The potential for *in vivo* contrast enhancement capabilities of a radical dendrimer specially designed to act as a *T*_1_ contrast agent for MRI was described in GL261 orthotopic GB-bearing mice. In particular, the third generation of a water-soluble radical dendrimer family based on poly(phosphorhydrazone) dendrimers fully functionalized with PROXYL radicals on the periphery (G3-Tyr-PROXYL-ONa radical dendrimer), presenting high *r*_1_ relaxivity (13 mM^–1^·s^–1^). MR-based biodistribution studies showed contrast enhancement mostly in the kidney cortex and pelvis, suggesting the relevance of renal excretion for this compound, confirmed by EPR analyses of the selected organs. Remarkably, it provides suitable contrast enhancement on murine GL261 glioblastoma tumors comparable to commercial Gd-based contrast agents (0.1 mmol/kg), at a 4 times lower concentration (0.025 mmol/kg dose), mitigating concerns about toxic metal accumulation. In fact, no signs of toxicity or weight loss were detected in mice after systemic intravenous tail-vein injection of the radical dendrimer. In addition, the selective accumulation of the G3 radical dendrimer in brain tumor tissue is a relevant and desirable characteristic in a contrast agent to be used for brain tumors. The G3 radical dendrimer also exhibited longer retention within the tumor, which allows tumor imaging over longer time frames (≥2.5 h) than Gd chelates which present faster clearance profiles. Moreover, high stability of the radicals anchored on the dendrimer surface when subjected to biological media has been demonstrated both *ex vivo* and *in vivo*. By EPR, it proved to be much higher (≥15 h) than that of isolated nitroxides, which are rapidly reduced (half-lives on the order of minutes).

Therefore, the high contrast enhancement (high relaxivity) and high stability of the G3 radical dendrimer species demonstrate that the two major limitations of nitroxyl radicals have been overcome, even in *in vivo* conditions. At the same time, it may also solve the major concern of Gd-based CAs, their established toxicity. These important features, together with the selective accumulation in brain tumor tissues and the longer imaging time frames in comparison to Gd-based CA, make the G3-Tyr-PROXYL-ONa radical dendrimer a viable alternative to metal-based MRI contrast agents, particularly on MRI analysis of glioblastomas.

## Supporting Information Available

Characterization of G3-Tyr-PROXYL-ONa, sequential axial *T*_1_w MRI acquired along time after *ex vivo* stereotactic injection of the G3 dendrimer, *T*_1_ estimation from *T*_1_ map sequences from *ex vivo* MRI acquisitions, EPR spectra of the G3 radical dendrimer prior to be injected and after injection (from the collected urine), additional EPR-based biodistribution data, DCE-MRI of tumor-bearing mouse administered with 0.00625 mmol/kg, and *T*_1_w MRI for follow-up of tumor contrast enhancement after G3 administration to tumor-bearing mouse at 0.025 mmol/kg (PDF)
