# Supplementary material for: Metal-Free Radical Dendrimers as MRI Contrast Agents for Glioblastoma Diagnosis: Ex Vivo and In Vivo Approaches
Source: Biomacromolecules. 2022 Jun 24;23(7):2767–77. doi: 10.1021/acs.biomac.2c00088 (PMC9277593; doi:10.1021/acs.biomac.2c00088)
Supplement: Supplementary file 1 — bm2c00088_si_001.pdf [file bm2c00088_si_001.pdf]

Supporting Information

Metal-free radical dendrimers as MRI contrast agents for glioblastoma diagnosis: ex vivo and in vivo approaches

Songbai Zhang,[a] Vega Lloveras,[a,b,*] Silvia Lope,[c,b] Pilar Calero-Pérez,[d,b] Shuang Wu,[d] Ana Paula Candiota,[b,d,e,*] and José Vidal-Gancedo[a,b,*]

[a]*Institut de Ciència de Materials de Barcelona, ICMAB–CSIC, Campus UAB, 08193 Bellaterra, Spain*
[b]*CIBER de Bioingeniería, Biomateriales y Nanomedicina, Instituto de Salud Carlos III, Campus UAB, 08913 Bellaterra, Spain*
[c] *Servei de Ressonància Magnètica Nuclear, Universitat Autònoma de Barcelona, 08193 Bellaterra, Spain*
[d] *Departament de Bioquímica i Biologia Molecular, Unitat de Bioquímica de Biociències, Edifici Cs, Universitat Autònoma de Barcelona, 08193 Bellaterra, Spain.*
[e] *Institut de Biotecnologia i de Biomedicina (IBB), Universitat Autònoma de Barcelona, 08193 Bellaterra, Spain*

Characterization of G3-Tyr-PROXYL-ONa radical dendrimer. The G3-Tyr-PROXYL-ONa radical dendrimer was characterized quantitatively by EPR and size exclusion chromatography (SEC). A quantitative EPR analysis of G3-Tyr-PROXYL-ONa was done in the same conditions than the previously reported family of Gn-Tyr-PROXYL-OLi derivatives[1] (Figure S1a). The calculated double integral value matched perfectly with that obtained with the G3-Tyr-PROXYL-OLi derivative (Table S1) supporting the 48 theoretical number of PROXYL units present in the structure. SEC chromatography confirmed the purity of G3-Tyr-PROXYL-ONa radical dendrimer (Figure S1b).

a)

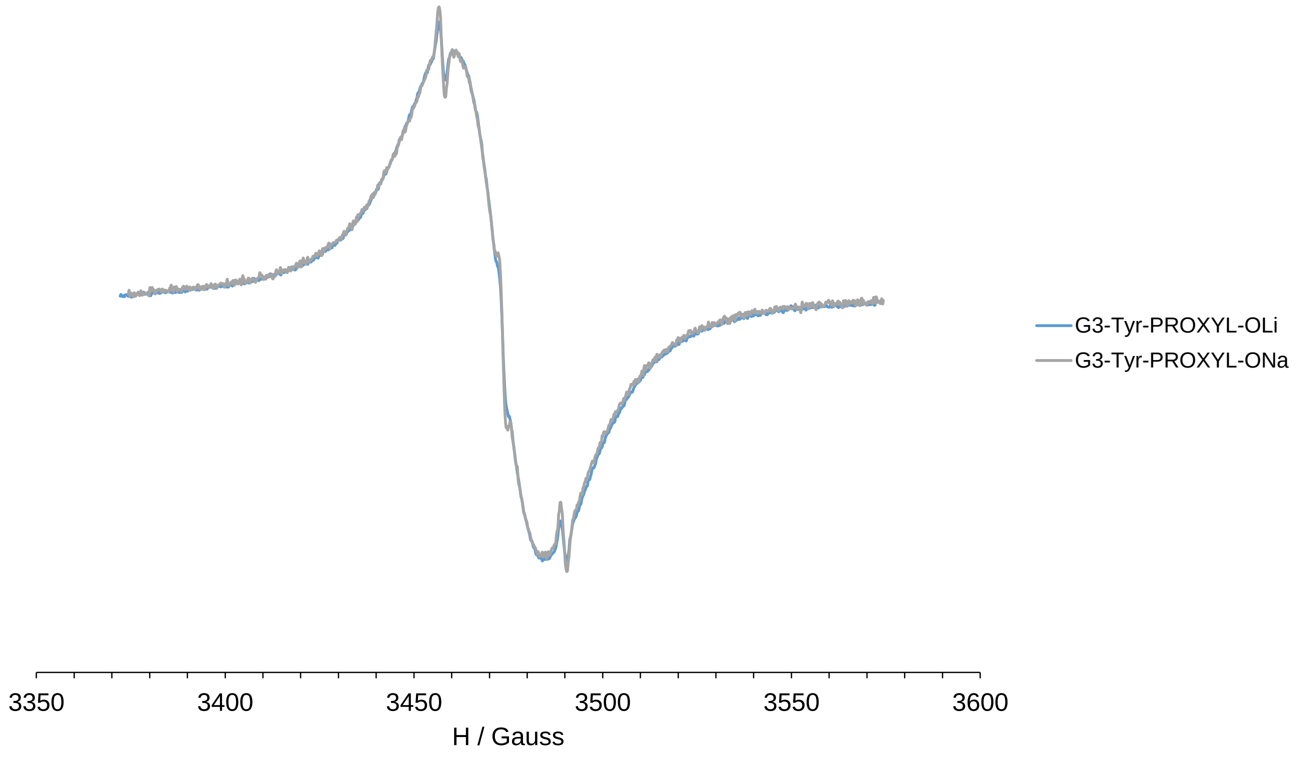

b)

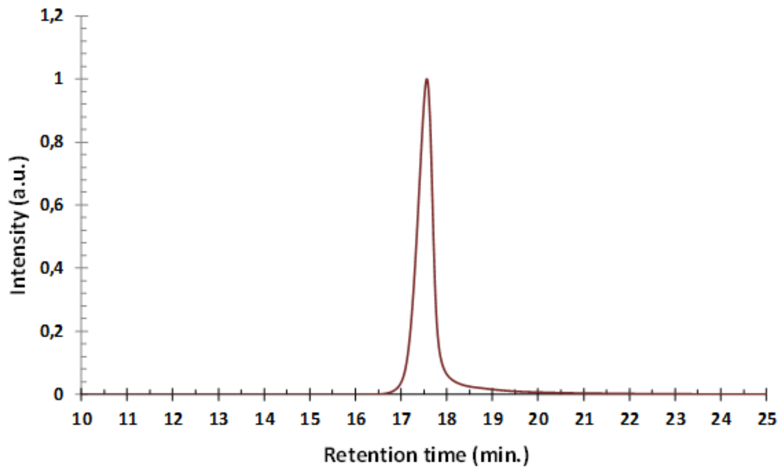

Figure S1. a) EPR spectra of G3-Tyr-PROXYL-OLi and G3-Tyr-PROXYL-ONa radical dendrimers at 300 K and 0.21 mM, in water. b) SEC chromatogram of G3-Tyr-PROXYL-ONa radical dendrimer in water with LiCl 0.25 mM.

Table S1. Double integral values of the EPR spectra of G3-Tyr-PROXYL-OLi and G3-Tyr-PROXYL-ONa at 300 K and 0.21 mM.

Radical dendrimer	Double Integration/10^6 (a.u.)
G3-Tyr-PROXYL-OLi	76±4
G3-Tyr-PROXYL-ONa	77±4

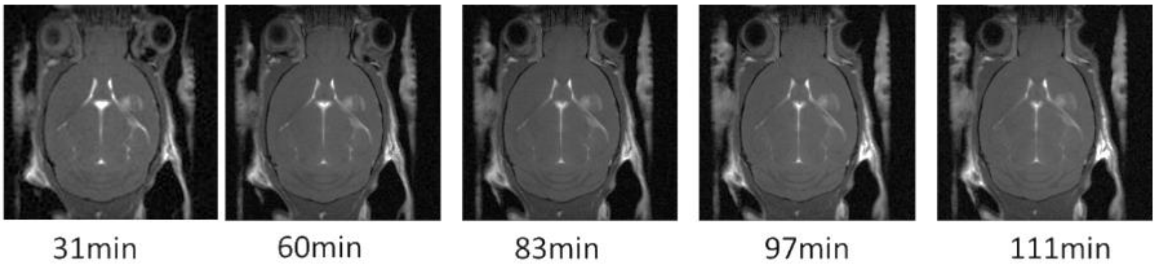

Figure S2. Sequential axial T1w MRI acquired along time after *ex vivo* stereotactic injection of G3 dendrimer (1.25 nmol at each injection point). The time after the first injection is shown below each MRI. Minutes are not exact multiples because other MRI sequences were interleaved between acquisitions.

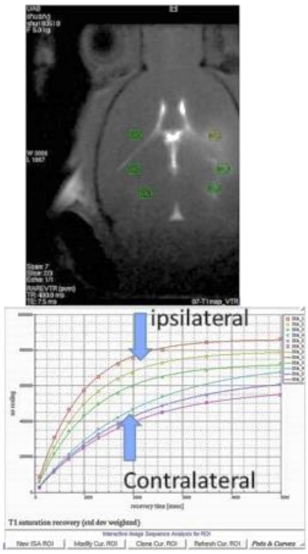

Figure S3. T_1 estimation from T_1 map sequences with Image Sequence Analysis (ISA) package tool from Paravision 5.0 and example of adjustments done for different ROIs. Ipsilateral points presented shorter T_1 in comparison with contralateral ROIs analyzed. Data from *ex vivo* MRI acquisitions.

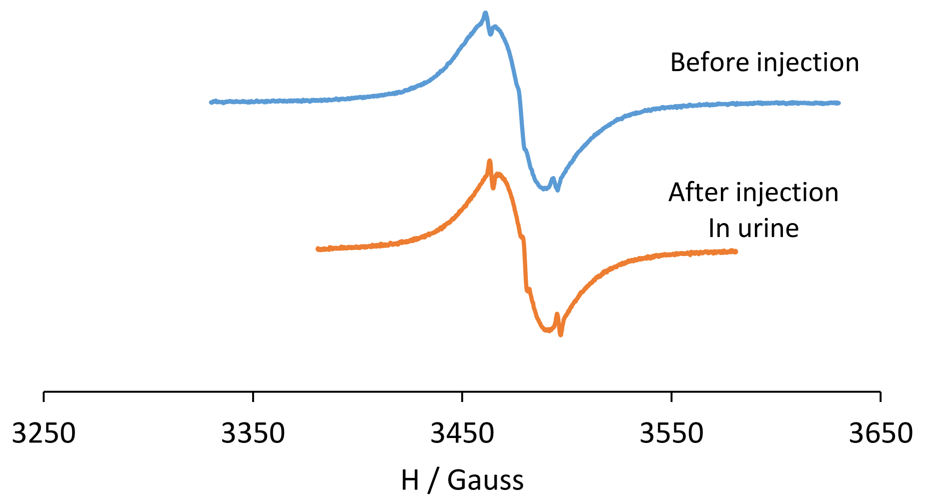

Figure S4. EPR spectrum from urine (diluted 1:2) of a mouse administered with 0.00625 mmol/Kg of G3 radical dendrimer, euthanized after biodistribution assays, compared with the spectrum of the initial radical dendrimer solution prior to be injected.

Additional EPR-based biodistribution data

A mouse administered with the lower dose (0.00625 mmol/Kg) was euthanized and chosen organs (urine, kidneys and liver) were studied by electron paramagnetic resonance (EPR) (Figure 6 from the manuscript). A similar mass of kidney and liver tissues was analyzed by EPR. For urine analysis, the collected urine was diluted 1:2 with miliQ water to get the optimum volume to be measured in a flat cell (a special EPR cell for aqueous solutions).

We were able to determine the concentration of G3 radical dendrimer in the urine by EPR, by comparison with the spectral area of pure compound spectra at known concentrations. Taking into account the dilution to ½ previously mentioned, the number of calculated mols in the urine was around 30 nmol, that means an approximately 20% of the total amount injected intravenously, after ca. 1.5 h of injection.

On the other hand, the EPR spectra shape of kidneys and liver is totally different from that of urine. Since the radical dendrimer is not found in liquid conditions (isotropic conditions) but within a dense tissue of an organ, i.e. under anisotropic conditions, the spectral shape corresponds to the slow-motion regime, close to the rigid limit. In these cases, we cannot discard the inactivation of some radicals of the dendrimer, but the observation of radical dendrimer EPR signal beyond 1.5 h after injection also confirms its stability *in vivo*.

We also analyzed the kidneys, liver, brain tumor, healthy brain, and muscle of a GL261-tumor-bearing mouse administered with the same dose 0.00625 mmol/kg 1.5 h postinjection (Figure S5). A similar mass of the chosen organs (about 47 mg each) was weighed.

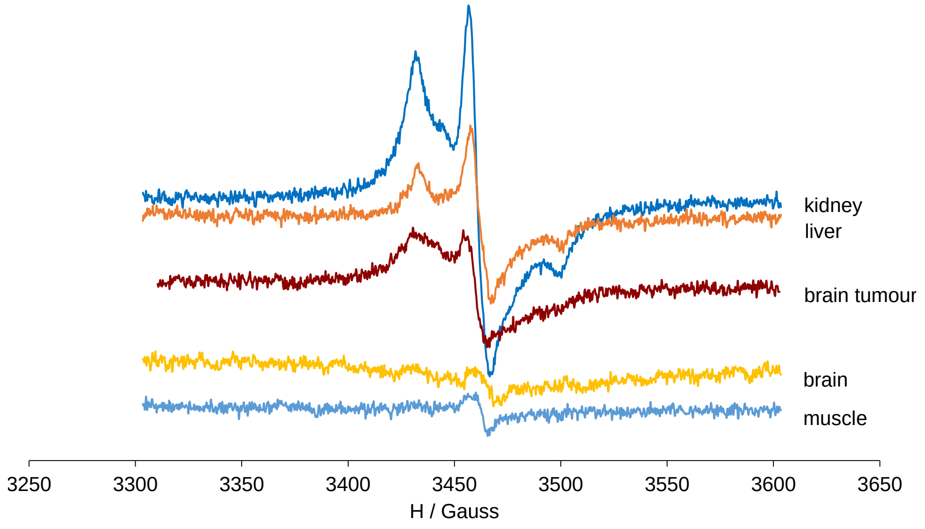

Figure S5. EPR spectra of kidneys, liver, brain tumor, contralateral healthy brain, and muscle from GL261-tumor-bearing mice 1.5 h after administration of 0.00625 mmol/kg of the G3 radical dendrimer.

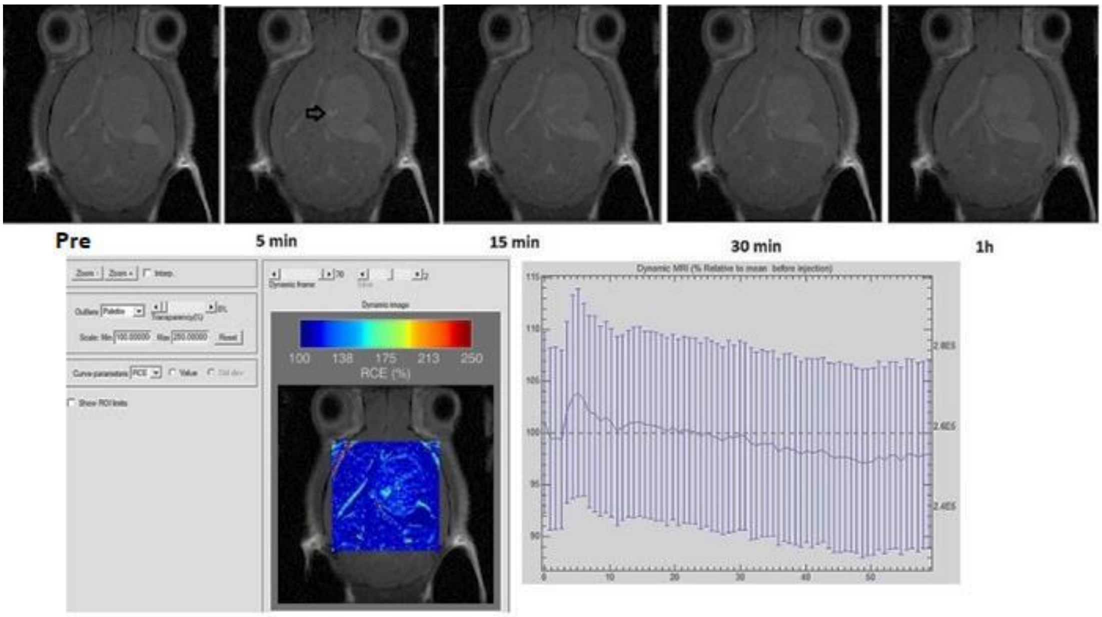

Figure S6. Top: Tumor bearing mouse #2 administered with 0.00625 mmol/Kg of the G3 radical dendrimer. A slight enhancement was seen in tumor border (black arrow) with *in vivo* MRI acquisitions. Bottom: DCE-MRI of the same mouse showing color-code scale for a chosen ROI and kinetics evolution. The enhancement in tumor border is confirmed in the RCE coloured map.

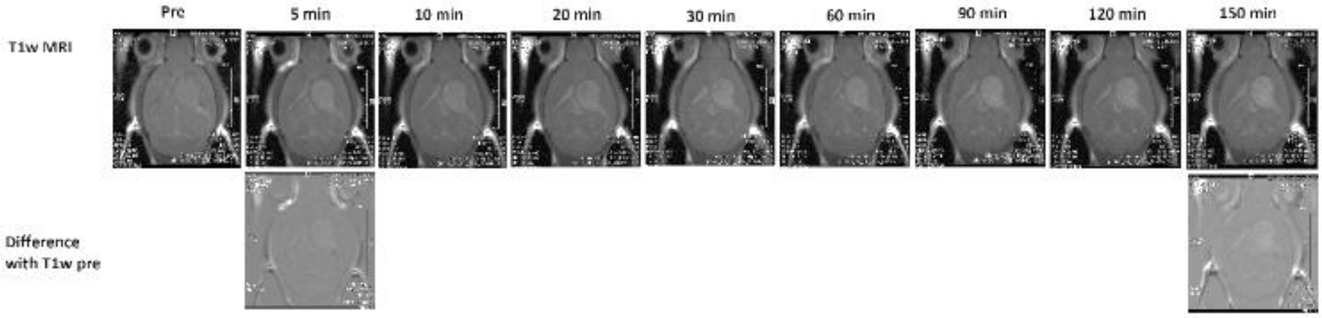

Figure S7. Top: T1w MRI for follow-up of tumor contrast enhancement after G3 administration to tumor-bearing mouse #3 at 0.025 mmol/Kg. Bottom: difference images after 5 minutes and 2.5 h of administration.

---

[1] Pinto, L.F.; Lloveras, V.; Zhang, S.; Liko, F.; Veciana, J.; Muñoz-Gómez, J.L.; Vidal-Gancedo, J. Fully Water-Soluble Polyphosphorhydrazone-Based Radical Dendrimers Functionalized with Tyr-PROXYL Radicals as Metal-Free MRI T1 Contrast Agents. *ACS Appl. Bio Mater.* **2020**, *3*, 369–376.
